# lncRNA ELFN1-AS1 enhances the progression of colon cancer by targeting miR-4270 to upregulate AURKB

**DOI:** 10.1515/med-2022-0582

**Published:** 2022-12-09

**Authors:** Shuangqin Peng, Yanjun Luo, Lijuan Chen, Kang Dai, Qin Wang

**Affiliations:** Department of Pediatrics, Maternal and Child Hospital of Hubei Province, Tongji Medical College, Huazhong University of Science and Technology, Wuhan, 430070, Hubei, China; Department of Pathology, Maternal and Child Hospital of Hubei Province, Tongji Medical College, Huazhong University of Science and Technology, No. 745 Wuluo Road, Hongshan District, Wuhan, 430070, Hubei, China; R&D Department, Wensheng Biotechnology Co., Ltd., Wuhan 430000, Hubei, China

**Keywords:** colon cancer, lncRNA ELFN1-AS1, miR-427, AURKB

## Abstract

The oncogenic role of lncRNA ELFN1-AS1 has been described in different cancers, including colon cancer (CC). However, how ELFN1-AS1 regulates CC malignancy remains unclear. In this study, ELFN1-AS1, AURKB, and miR-4270 expression levels in CC cells and tissues were determined using RT-qPCR and western blotting. CCK-8 and wound healing assays were also performed to analyze alterations in CC cell proliferation and migration. The expression of apoptosis-related proteins (Bax and Bcl-2) was determined via western blot analysis. RNA immunoprecipitation (RIP) assays coupled with luciferase reporter assays were employed to verify the relationship between miR-4270, ELFN1-AS1, and AURKB. An *in vivo* assay was performed using xenograft tumors in mice to detect the change of tumor growth. It was found that AURKB and ELFN1-AS1 expression was upregulated, whereas miR-4270 was downregulated in CC cells and tissues. ELFN1-AS1 silencing exhibited anti-proliferative, anti-migratory, and pro-apoptotic effects in CC cells. The tumor-suppressive effect of ELFN1-AS1 silencing was verified using *in vivo* assays. MiR-4270 was predicted to be a target of ELFN1-AS1 and AURKB as a target of miR-4270. Their interactions were further elucidated using luciferase reporter and RNA RIP assays. More importantly, treatment with a miR-4270 inhibitor not only rescued the tumor-suppressing effect of ELFN1-AS1 silencing but also abrogated the tumor suppressor functions of AURKB silencing in CC cells. Taken together, the ELFN1-AS1/miR-4270/AURKB axis facilitates CC tumorigenesis; therefore, targeting this axis might be a promising intervention in preventing CC progression.

## 1 Introduction

Colon cancer (CC) is the fourth most frequently diagnosed cancer and a predominant cause of cancer-related deaths worldwide. It has been reported that 1,148,515 new CC cases were diagnosed globally in 2020 [1]. Cancer-related fatality resulting from CC ranks behind lung, breast, stomach, and liver cancers, accounting for approximately 5% of all cancer-related mortalities [1]. Furthermore, CC could occur at any age, even in infancy [2,3]. Due to the asymptomatic or paucisymptomatic nature of the disease, most patients with CC are already diagnosed at a late stage upon initial clinical visit [4]. Notably, clinicians easily misdiagnose and miss CC in children or adolescents because these patients cannot detail their disease history and irregular living habits [3]. Although enormous improvements in therapies have been made in the last few years, the 5-year survival rate of patients with advanced CC is less than 5% [5]. In the era of precision medicine, appropriate therapeutic targets can be identified by revealing the genetic characteristics underlying cancer development [6]. Therefore, deciphering the mechanisms underlying CC progression is needed to facilitate diagnosis and therapy for this disease, especially in children.

Although the human genome is pervasively transcribed, >98% of RNA transcripts do not possess coding capacity. Among these, long noncoding RNAs (lncRNAs) are a subtype of transcripts longer than 200 bp. Compelling evidence has demonstrated that lncRNAs facilitate multiple cellular functions such as proliferation and migration. Abnormalities in these vital cellular functions have been described in various human disorders, including cancers [7,8]. An increasing number of lncRNAs are being discovered to play a role in CC progression. For example, the lncRNA LINC00662 promotes the expression of the ERK signaling pathway, facilitating CC progression [9]. Using data obtained from the TCGA database, Lin et al. identified immune-related lncRNAs, which are associated with the clinicopathological features of patients with CC [10]. Targeting the lncRNA DNAJC3-AS1 attenuates the migration, invasion, and epithelial–mesenchymal transition of CC cells [11]. ELFN1-AS1 is a newly identified lncRNA located on the human chromosome 7p22.3. The oncogenic role of lncRNA ELFN1-AS1 has been described in different cancers, including CC [12–16]. However, the regulatory mechanisms underlying CC progression remain unknown.

MicroRNAs (miRNAs) are 18–22 nucleotide-lncRNAs that inhibit RNA translation, promote RNA degradation, and regulate transcription and splicing processes [17]. The role of miRNAs in cancer has been studied previously. For example, miR-122, miR-1271, and miR-15b were associated with the risk of renal clear cell carcinoma [18,19]. A growing body of evidence suggests that several miRNAs are differentially expressed in CC and play important roles in its diagnosis and progression. MiR-378 acts as a tumor suppressor in CC as it has been found to be low in CC tissues and cell lines and inhibits proliferation, migration, and invasion of CC cells by targeting SDAD1 [20]. On the contrary, miR-510 acts as a tumor promoter in CC as it has been found to be considerably increased in CC tissues and cell lines and has been shown to promote CC cell proliferation, migration, and invasion by targeting SRCIN 1 [21]. Furthermore, the upregulation of miR-654-5p and downregulation of miR-376b-3p might be related to tumor progression in CC [22]. Moreover, lncRNAs have been found to epigenetically, transcriptionally, or post-transitionally modulate gene expression, contributing to their functional diversity [23]. lncRNAs negatively regulate miRNA expression, owing to their promiscuity with miRNA seed sequences, which is recognized as a competing endogenous RNA (ceRNA) mechanism. For instance, LINC00662 functions as a ceRNA of miR-340-5p, stimulates the ERK signaling pathway, and promotes CC progression [9]. The oncogenic NF-κB signaling pathway is hyperactivated by a ceRNA network consisting of the lncRNA DNAJC3-AS1, miR-214-3p, and LIVIN, resulting in CC progression [11]. Examples of ELFN1-AS1 ceRNA activity in different cancers include sponging miR-183-3p [24], miR-497-3p [25], mi-4644 [14], and miR-2470 [15]. Feng et al. found that miR-4270 was a target of ELFN1-AS1 in retinoblastoma (RB) [15]. Interestingly, miR-4270 acts as a tumor suppressor or oncomiR in various cancers [26,27]. However, the role of miR-4270 in CC has not yet been elucidated.

In this study, we investigated the role of the lncRNA ELFN1-AS1 in CC *in vitro* and *in vivo*. We also explored the ceRNA activity of ELFN1-AS1 on miR-4270 in CC. Our findings suggest that inhibiting ELFN1-AS1 expression might represent a therapeutic target in CC, implicating ELFN1-AS1 silencing as a promising therapeutic intervention in patients with CC.

## 2 Materials and methods

### 2.1 Patients and tissue samples

In total, 45 pairs of CC tissues and corresponding noncancerous tissues were obtained from the Maternity and Child Healthcare Hospital in Hubei, China. The patients whose samples were taken did not receive preoperative chemotherapy or chemoradiotherapy. After dissection and pathological confirmation, the clinical specimens were immediately frozen in liquid nitrogen and stored at –80°C until further analysis.


**Ethical approval:** Ethics Committee of the Maternal and Child Hospital of Hubei Province (Wuhan, China) approved this work. All clinical samples were processed in strict concurrence with the Declaration of Helsinki s ethical standards. Informed consent was obtained from each patient before the tumors were surgically resected.

### 2.2 Cell culture

Normal colon FHC and CC cell lines (SW480, HCT116, and SW620) were obtained from the American Type Culture Collection (USA). FHC cells (DMEM, Thermo Fisher, USA), HCT116 (McCoy’s 5A Medium, Thermo Fisher), SW480, and SW620 cells (Leibovitz’s L-15 Medium, Thermo Fisher) were maintained in the indicated culture media supplemented with FBS (Thermo Fisher) and 1% penicillin–streptomycin solution (Thermo Fisher). The cells were cultured at 37°C in an incubator with an atmosphere of 5% CO_2_.

### 2.3 Cell transfection

Si-ELFN1-AS1 (si-lnc-1 and si-lnc-2), sh-ELFN1-AS1, si-AURKB, miR-4270 inhibitor/mimic, and their corresponding NCs were obtained from Hanbio Shanghai Biotechnology Co. Ltd, China. The synthesized oligonucleotides were transfected into 80% confluent SW480 and SW620 cell cultures using Lipofectamine 3000 reagent (Invitrogen, USA). After 48 h, RT-qPCR was conducted to analyze the transfection efficiency. To establish stable ELFN1-AS1-knockdown cells, sh-NC and sh-ELFN1-AS1 lentivirus vectors were introduced into SW480 cells, followed by neomycin selection. Five weeks later, neomycin-surviving cells were collected and validated using RT-qPCR.

### 2.4 CCK-8 assay

SW480 and SW620 cells were maintained in 96-well plates (5,000 cells per well) for 0, 24, 48, and 72 h. Next, 10 μL of CCK8 reagent (Thermo Fisher) was added to the cells and incubated for another 3 h. The absorbance of each well was then measured at 450 nm using a spectrophotometer.

### 2.5 Wound-healing assay

Briefly, 2  ×  10^5^ SW480 and SW620 cells were seeded in each well of 24-well plates and were cultured until the cell monolayers reached 90% confluence. Next, a new 1-mL pipette tip was used to scratch the cell monolayer, and the scratched cellular debris was washed away. After incubation for another 24 h, the cells were photographed under a microscope at 100× magnification.

### 2.6 RT-qPCR

A RiboZol RNA kit (AMRESCO, USA) was used to extract total RNA from CC cells and tissues. Reverse transcription from isolated total RNA (3 μg) was conducted using a Titan One Tube RT-PCR kit (Merck, USA) or HyperScript III miRNA 1st Strand cDNA Synthesis Kit (NovaBio, China), according to the respective manufacturer’s instructions. Premixed maximum SYBR Green/ROX qPCR (Thermo Scientific, USA) was applied for qPCR analysis on the StepOnePlus system (Applied Biosystems, USA). The relative expressions of the target genes were calculated using the 2^∆∆Ct^ method and were normalized using U6 or GAPDH as an internal control. The sequences of the primers used are listed in Table 1.

**Table 1:** Real-time PCR primer synthesis list

Gene	Sequences	
ELFN1-AS1	Forward	5′−TGTCATTCACTCCGAGACGC−3′
	Reverse	5′−TGAGAGTGAATTCGGGGTGC−3′
miR-4270	Forward	5′−ACAAATAGCTTCAGGGAGTCAGG−3′
	Reverse	5′−GACCCACTTTCTTCCCAGCA−3′
AURKB	Forward	5′−GGGAGAGTAGCAGTGCCTTG−3′
	Reverse	5′−CAGCTCTTCTGCAGCTCCTT−3′
U6	Forward	5′−CTCGCTTCGGCAGCACA−3′
	Reverse	5′−AACGCTTCACGAATTTGCGT−3′
GAPDH	Forward	5′−AGAAAAACCTGCCAAATATGATGAC−3′
	Reverse	5′−TGGGTGTCGCTGTTGAAGTC−3′

A nuclear and cytoplasmic protein extraction kit (Beyotime, China) was used for the nucleocytoplasmic fractionation of SW480 and SW620 cells. Both extracts were subjected to RT-qPCR.

### 2.7 Western blot

Radioimmunoprecipitation assay lysis buffer was used to prepare SW480 and SW620 cell lysates. After microcentrifugation, the protein samples were quantified using a BCA Protein Assay kit (Abcam, USA). Protein samples (20 µg) were separated using 10% SDS-PAGE and transferred to a PVDF membrane at a constant voltage of 75 V. The membranes were blocked with 5% skim milk at room temperature for 1 h and then incubated with anti-AURKB (Cat#:36-5200; 1:1,000; Thermo Fisher), anti-Bax (Cat#:33-6400; 1:1,000; Thermo Fisher), anti-Bcl-2 (Cat#: MA5-11757; 1:1,000; Thermo Fisher), and anti-GAPDH antibodies (Cat#: MA1-16757; 1:1,000; Thermo Fisher) overnight at 4°C. The next day, the membranes were incubated at room temperature with HRP-conjugated goat anti-rabbit/mouse secondary antibodies (Cat#: SA5-10204 or A32727; 1:1,000; Thermo Fisher) for 2 h. The blots were then developed using BeyoECL Plus mix substrate (Beyotime, USA), and ImageJ (Image J Software, National Institutes of Health, USA) was used to quantify the target proteins.

### 2.8 Murine xenograft model

BALB/c mice (6–8-week old, 20–25 g) were obtained from the Animal Resource Center at the Wuhan Institute of Virology, Chinese Academy of Sciences, China. Mice were caged individually under specific pathogen-free conditions with good ventilation. Food and water were supplied daily. To construct a CC xenograft mouse model, SW480 cells were stably transfected with sh-ELFN1-AS1 or sh-NC and harvested in the exponential growth phase. Next, the mice received subcutaneous injections (1 × 10^7^ cells/mouse) of the prepared CC cells. Xenograft size was monitored every 4 days using calipers (length, width, and height). The mice were anesthetized and euthanized via CO_2_ asphyxiation on the 28th day. Finally, the tumors were harvested and weighed.


**Ethical approval:** The procedures executed in the animal study were authorized by the Maternal and Child Hospital of Hubei Province. All experiments on animals comply with the ARRIVE guidelines.

### 2.9 Luciferase reporter assay

The wild-type (WT) sequences of miR-4270 binding to the 3′-UTR of ELFN1-AS1 or AURKB and the corresponding designed mutant (MUT) sequences were inserted into the GP-mirGLO Dual-Luciferase miRNA Target Expression Vector (Promega, USA). The luciferase vectors containing ELFN1-AS1-WT, ELFN1-AS1-MUT, AURKB-WT, or AURKB-MUT were co-incubated with a miR-4270 or NC mimic in SW480 and SW620 cells. After 48 h, luminescence was monitored using a dual-luciferase reporter assay system (Promega). The outcomes are presented as normalized luciferase activity (Renilla luciferase/firefly luciferase).

### 2.10 RNA immunoprecipitation (RIP) assay

An EZ-Magna RIP Kit (Millipore) was employed as per the supplier’s directions to confirm the direct binding of ELFN1-AS1 and miR-4270. Briefly, SW480 and SW620 cells were lysed using a lysis buffer. The collected lysates were mixed with magnetic beads pre-covered with anti-IgG or anti-AGO2 antibodies. Finally, RT-qPCR was performed to evaluate the expression of the immunoprecipitated complex.

### 2.11 Statistical analysis

Statistical analyses were performed using GraphPad Prism 9 software (Prism, USA). The results are reported as mean ± SD. Student’s *t*-test was performed for two-group comparisons, while one-way analysis of variance (ANOVA) with Tukey’s *post-hoc* analysis was performed for multiple-group comparisons. The correlation between miR-4270 and the lncRNAs ELFN1-AS1 or AURKB was analyzed using Pearson’s correlation analysis. *P* < 0.05 was considered statistical significance.

## 3 Results

### 3.1 Upregulation of the cytoplasmic lncRNA ELFN1-AS1 in CC

**Figure 1:**
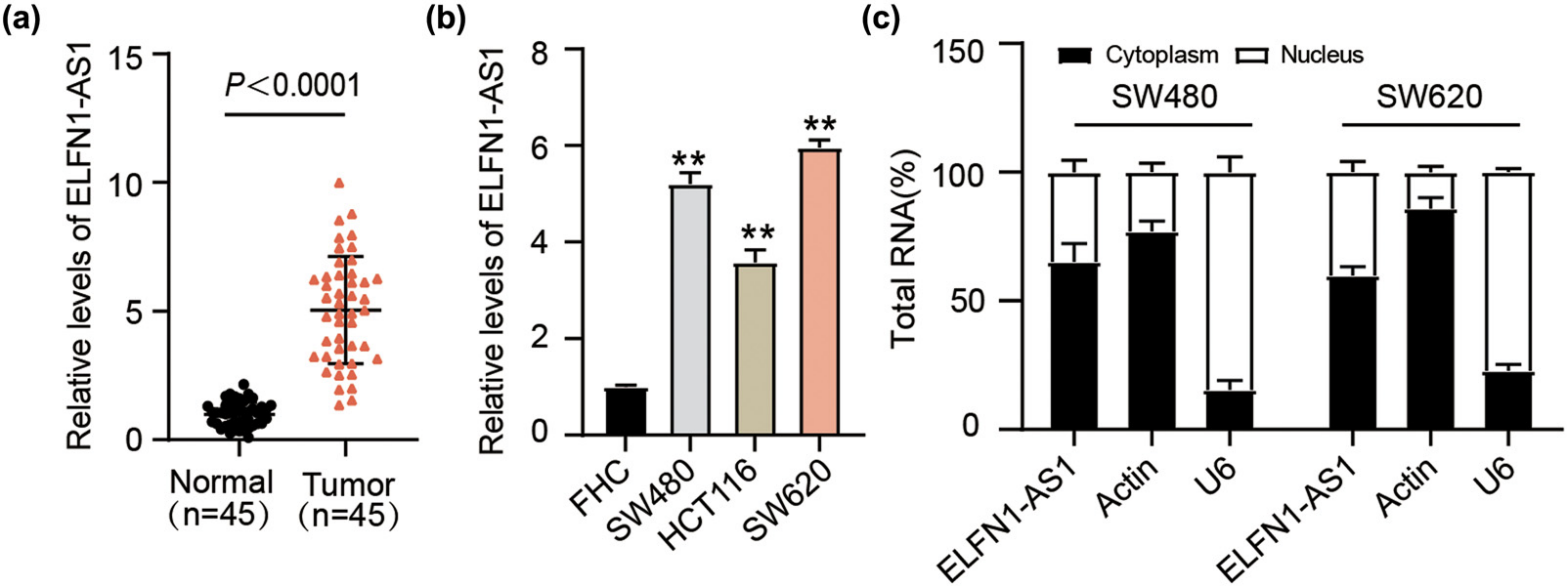
Upregulation of the cytoplasmic lncRNA ELFN1-AS1 in CC. (a) RT-qPCR of ELFN1-AS1 expression in CC tissues and normal tissues. (b) RT-qPCR of ELFN1-AS1 expression in CC cells (SW480, HCT116, and SW620). **P＜0.01 vs FHC. (c) RT-qPCR analysis of nuclear and cytoplasmic ELFN1-AS1 in both SW480 as well as SW620 cells.

To determine the role of ELFN1-AS1 in CC, its expression in 45 pairs of colon tissues and corresponding noncancerous tissues was examined. RT-qPCR results showed that ELFN1-AS1 expression was higher in tumor tissues than in normal tissues (Figure 1a). Higher ELFN1-AS1 levels were also detected in CC cells (SW480, HCT116, and SW620) than in normal FHC cells (Figure 1b). In particular, an increase in ELFN1-AS1 expression was observed in SW480 and SW620 cells compared to FHC cells. Meanwhile, the cytoplasm/nucleus separation assays showed that ELFN1-AS1 was predominantly enriched in the cytoplasm of SW480 and SW620 cells (Figure 1c), suggesting that ELFN1-AS1 exerts important cytoplasmic functions, such as acting as a miRNA sponge.

### 3.2 ELFN1-AS1 silencing retards CC growth *in vitro* and *in vivo*

**Figure 2:**
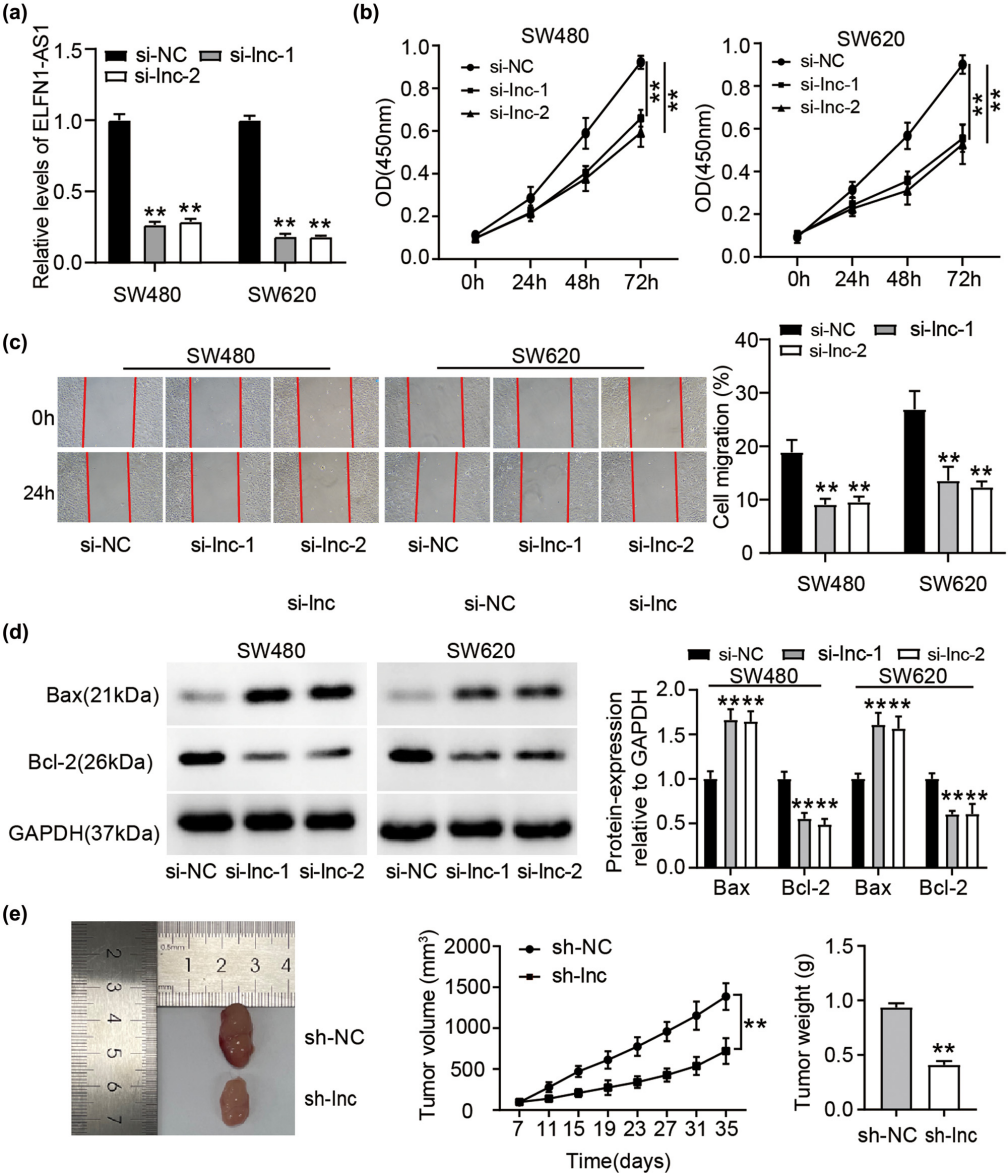
ELFN1-AS1 silence retards CC growth *in vitro* and *in vivo*. si-ELFN1-AS1 and si-NC were transfected into SW480 and SW620 cells. After 48 h, (a) ELFN1-AS1 expression in si-ELFN1-AS1 (si-lnc-1 and si-lnc-2) or si-NC transfected SW480 as well as SW620 cells was determined through RT-qPCR. **P＜0.01 vs si-NC. (b) Quantification of cell proliferation of si-ELFN1-AS1 (si-lnc-1 and si-lnc-2) or si-NC transfected SW480 as well as SW620 was detected by CCK8 assays. **P＜0.01 vs si-NC. (c) Wound-healing assay of SW480 and SW620 transfected with si-ELFN1-AS1 (si-lnc-1 and si-lnc-2) compared to cells with or si-NC. Representative image shown. **P＜0.01 vs si-NC. (d) The Bax and Bcl-2 expression in SW480 and SW620 transfected with si-ELFN1-AS1 (si-lnc-1 and si-lnc-2) and si-NC was checked through western blotting. **P＜0.01 vs si-NC. (e) The *in vivo* ELFN1-AS1 silencing effect was evaluated in xenograft mouse models bearing tumors originating from SW480 cells. **P＜0.01 vs sh-NC.

To explore whether ELFN1-AS1 plays a role in CC tumorigenesis, we transfected si-ELFN1-AS1 (si-lnc-1 and si-lnc-2) into SW480 and SW620 cells. An obvious reduction in ELFN1-AS1 expression was observed in both si-lnc-1- and si-lnc-2-treated CC cells (Figure 2a). Moreover, si-lnc-1 and si-lnc-2 expression significantly decreased the viability of SW480 and SW620 cells (Figure 2b). Scratch assays also demonstrated that si-lnc-1 and si-lnc-2 treatment reduced the migration of both CC cell lines (Figure 2c). A clear apoptotic activation upon ELFN1-AS1 silencing (si-lnc-1 and si-lnc-2) in CC cells was evidenced via western blotting, which showed increased Bax and decreased Bcl-2 expression (Figure 2d). More interestingly, quantification of the size and weight of the xenograft tumors showed that ELFN1-AS1 silencing significantly reduced tumorigenesis *in vivo* (Figure 2e). Taken together, our findings suggested that the malignant behavior of CC cells was inhibited through ELFN1-AS1 silencing *in vitro* and impeded tumor growth *in vivo*.

### 3.3 ELFN1-AS1 targets miR-4270

**Figure 3:**
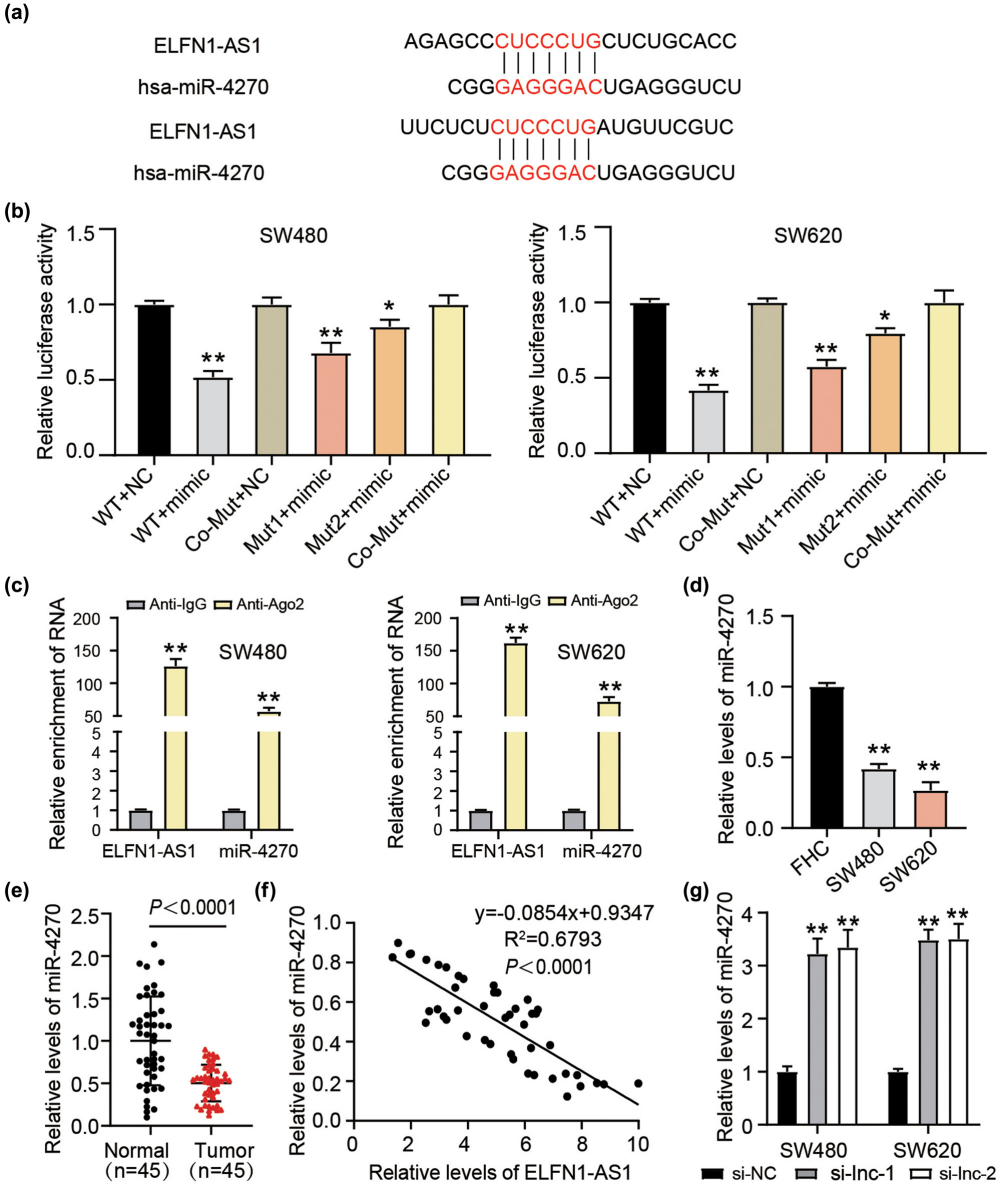
ELFN1-AS1 targets miR-4270. (a) Predicted lncRNA–miRNA interactions (ELFN1-AS1 and miR-4270) was predicted by bioinformatic analysis. (b) Luciferase activity in extracts from SW480 as well as SW620 cells co-transfected with ELFN1-AS1-related wide type or mutant luciferase vectors and miR-4270 mimic or NC. *P＜0.05, **P＜0.01 vs WT + NC. (c) RT-qPCR assessing AGO2 RIP by AGO2 antibody in RIP. **P＜0.01 vs Anti-IgG. (d) RT-qPCR analysis of miR-4270 expression in CC cells (SW480 and SW620) and FHC cells. **P＜0.01 vs FHC. (e) RT-qPCR analysis of miR-4270 expression in CC tissues and normal cells. (f) Pearson correlation coefficient between miR-4270 expression and ELFN1-AS1 expression. (g) miR-4270 expression in si-ELFN1-AS1 (si-lnc-1 and si-lnc-2) or si-NC transfected SW480 as well as SW620 cells was determined through RT-qPCR. **P＜0.01 vs si-NC.

Considering the cytoplasmic localization of ELFN1-AS1, we determined the potential downstream targets affecting ELFN1-AS1 function. Bioinformatic analysis showed that the ELFN1-AS1 sequence contained two putative binding sites for miR-4270 (Figure 3a). To validate this prediction, we fused the ELFN1-AS1 sequence and three mutated sequences to luciferase reporter vectors and then co-incubated them with a miR-4270 or NC mimic into SW480 and SW620 cells. Quantification of luciferase activity showed that the miR-4270 mimic remarkably reduced the transcriptional activity of ELFN1-AS1 or only partly influenced the transcriptional activity of Mut-1 and Mut-2 ELFN1-AS1; however, it failed to affect co-MUT transcription (Figure 3b). LncRNAs act as miRNA sponges, modulate miRNA activity, and form an RNA-induced silencing complex (RISC); the AGO2 protein is essential in this complex. Therefore, we performed AGO2 enrichment analysis on SW480 and SW620 cells to verify whether ELFN1-AS1 and miR-4270 could bind to the Ago2 protein in RISC. The results demonstrated that both ELFN1-AS1 and miR-4270 were enriched in the Ago2-IP fractions (Figure 3c). Therefore, miR-4270 is a potential target of ELFN1-AS. It is well-recognized that lncRNAs antagonize miRNA expression, weakening their action by binding to their binding site. The RT-qPCR analysis demonstrated that miR-4270 expression was downregulated in both CC cells and tissues (Figures 3d and 3e). A negative correlation existed between miR-4270 and ELFN1-AS1 levels in CC tissues (Figure 3f), while upregulated miR-4270 levels were observed in the si-lnc-1- and si-lnc-2-treated groups, as shown by the RT-qPCR results (Figure 3g). These data further validate the antagonizing interaction between miR-4270 and ELFN1-AS1.

### 3.4 ELFN1-AS1 targets miR-4270 and promotes malignant behaviors in CC cells

**Figure 4:**
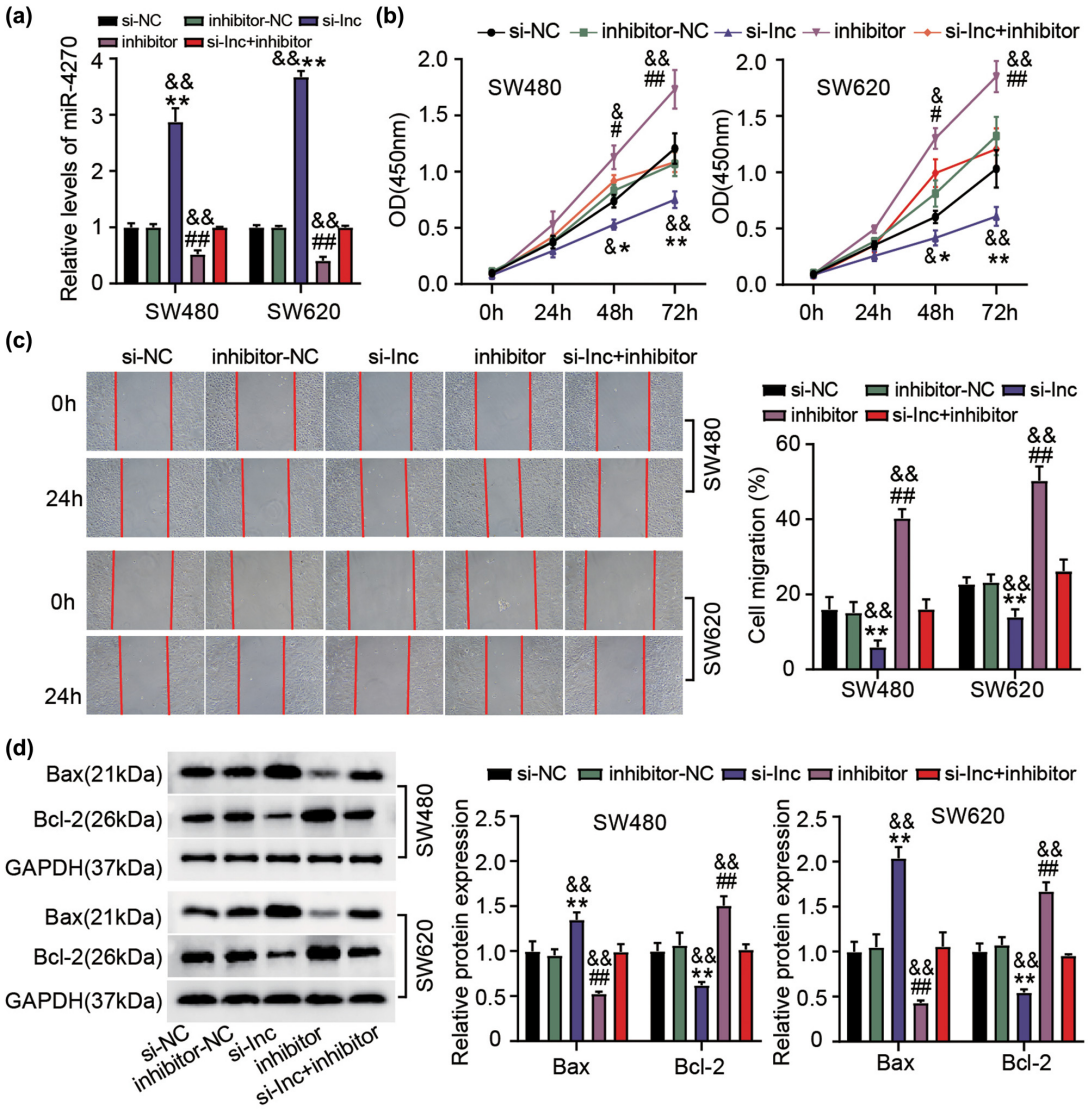
ELFN1-AS1 targeting miR-4270 promotes CC cell malignant behaviors. SW480 and SW620 cells was transfected with si-NC, si-ELFN1-AS1, miR-4270 inhibitor, inhibitor NC, and si-ELFN1-AS1 + miR-4270 inhibitor. After 48 h, (a) miR-4270 expression through RT-qPCR, (b) proliferation tested by CCK-8 assay, (c) migration measured with wound-healing assay. (d) The Bax as well as Bcl-2 expression in transfected SW480 and SW620 cells was checked through western blotting. *P＜0.05, **P＜0.01 vs si-NC; ^#^ *P*＜0.05, ^##^ *P*＜0.01 vs inhibitor-NC; ^&^ *P*＜0.05, ^&&^ *P*＜0.01 vs si-lnc + inhibitor.

We subsequently attempted to uncover if the interaction between ELFN1-AS1 and miR-4270 contributes to CC progression *in vitro*. Briefly, we co-incubated inhibitors of both si-ELFN1-AS1 and miR-4270 into SW480 and SW620 cells. The introduction of a miR-4270 inhibitor neutralized the increased expression of miR-4270 when co-incubated with si-ELFN1-AS in both these cell lines (Figure 4a). Next, we examined whether the miR-4270 inhibitor affected the malignant behavior of CC cells regulated by ELFN1-AS1 silencing. As described in Figure 4b, the blockade of miR-4270 resulted in an elevated proliferation of CC cells, which was almost nullified by ELFN1-AS1 silencing. Consistently, the increased migration of colon cells upon treatment with a miR-4270 inhibitor was abrogated by co-transfection with si-ELFN1-AS1 (Figure 4c). Additionally, resistance to apoptosis was observed in SW480 and SW620 cells treated with a miR-4270 inhibitor. This phenomenon was offset upon transfection of a miR-4270 inhibitor and si-ELFN1-AS1 (Figure 4d). These findings demonstrated that miR-4270 downregulation is required for the oncogenic activity of ELFN1-AS1.

### 3.5 MiR-4270 interacts with AURKB

**Figure 5:**
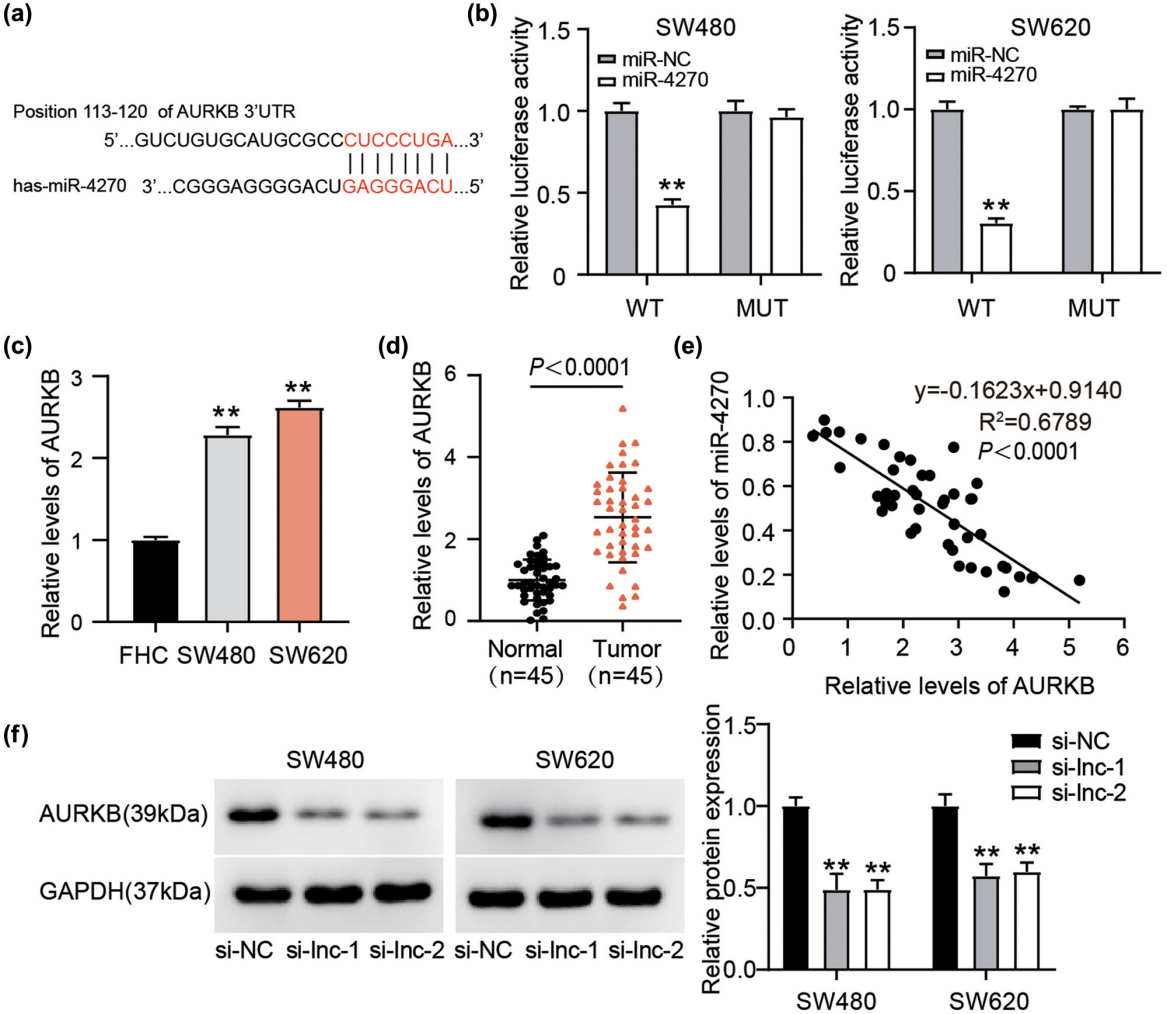
miR-4270 interacts with AURKB. (a) The miRNA-4270 binding sites in human AURKB 3′-UTR predicted through TargetScan. (b) Relative luciferase activity of AURKB 3′-UTR MUT and WT was determined after co-transfection with miR-4270 mimics or mimics NC. **P＜0.01 vs miR-NC. (c) RT-qPCR analysis of AURKB expression in CC cells (SW480 and SW620) and FHC cells. (d) RT-qPCR analysis of AURKB expression in CC tissues and normal tissues. (e) Pearson correlation analysis of miR-4270 expression with AURKB expression. (f) AURKB protein expression in si-ELFN1-AS1 (si-lnc-1 and si-lnc-2) or si-NC transfected SW480 as well as SW620 cells was determined through western blotting. **P＜0.01 vs si-NC.

Subsequently, we used TargetScan analysis to identify the potential targets of miR-4270. As predicted, the 3′-UTR of AURKB shared a complementary sequence with miR-4270 (Figure 5a). Direct binding between these molecules was also checked using luciferase reporter assays, which showed that the miR-4270 mimic disrupted 3′-UTR AURKB WT-mediated luciferase activity but had no effect on 3′-UTR AURKB MUT-mediated luciferase activity (Figure 5b). Furthermore, abundant AURKB expression was detected in both CC cells and tissues (Figures 5c and d). Their negative correlation further supports the idea that miR-4270 could be directly recognized by the 3′-UTR of AURKB (Figure 5e). Furthermore, western blot analysis showed that AURKB protein levels were lower in the si-lnc-1- and si-lnc-2-treated groups than in the si-NC-treated group (Figure 5f). Therefore, AURKB is a direct target of miR-4270 and is upregulated by ELFN1-AS1.

### 3.6 AURKB is required for the inhibitory effect of miR-4270 on the malignant behaviors of CC cells

**Figure 6:**
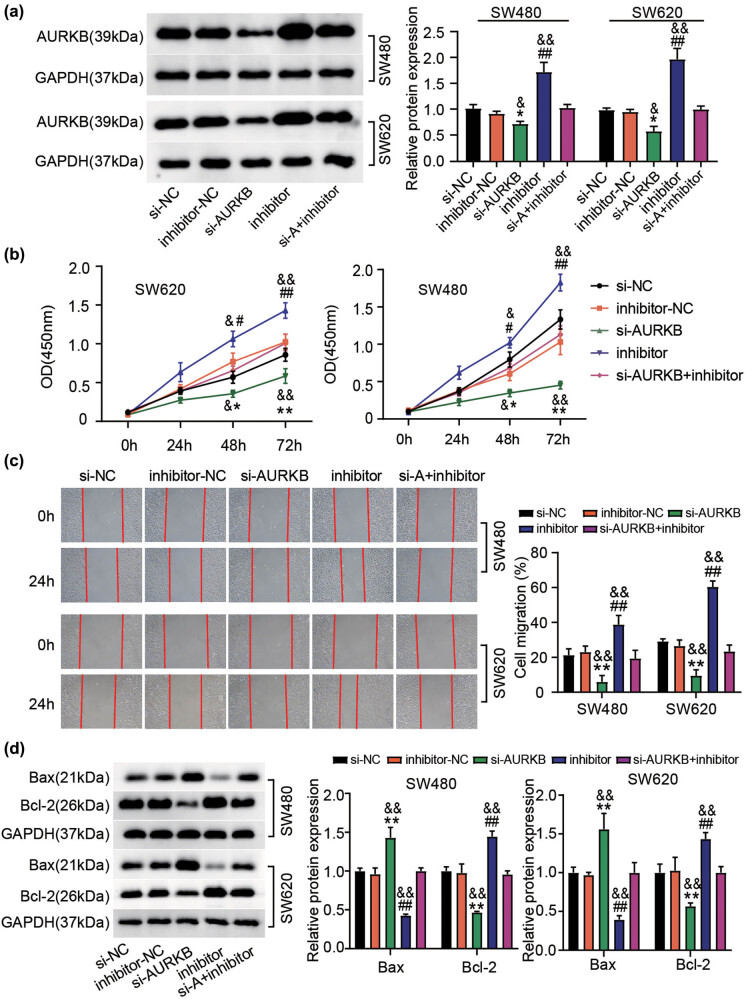
AURKB is required for the inhibitory effect of miR-4270 on CC cell malignant behaviors. SW480 and SW620 cells were transfected with miR-4270 inhibitor, inhibitor NC, si-AURKB, si-NC, and miR-4270 inhibitor + si-AURKB. 48 h later, (a) AURKB expression analysis through western blotting, (b) proliferation tested by CCK-8 assay, (c) migration measured with wound-healing assay. (d) Bax and Bcl-2 expression in transfected SW480 as well as SW620 cells was checked through western blot analysis. *P＜0.05, **P＜0.01 vs si-NC; ^#^ *P*＜0.05, ^##^ *P*＜0.01 vs inhibitor-NC; ^&^ *P*＜0.05, ^&&^ *P*＜0.01 vs si-AURKB + inhibitor.

Based on our initial findings, we then sought to determine whether AURKB expression dysregulation could explain the function of miR-4270 in CC. Hence, a miR-4270 inhibitor and si-AURKB were co-introduced into SW480 and SW620 cells. As shown in Figure 6a, treatment with the miR-4270 inhibitor enhanced AURKB expression, whereas this enhanced expression was almost nullified by si-AURKB treatment. Next, functional assays demonstrated that the loss of endogenous AURKB expression constrained CC cell viability and migration; however, this phenomenon was restored through the co-introduction of si-AURKB and a miR-4270 inhibitor (Figures 6b and 6c). Additionally, AURKB silencing induced the apoptotic activation of CC cells; however, this outcome was nullified by treatment with the miR-4270 inhibitor (Figure 6d). Taken together, our results indicate that miR-4270 inhibits the malignant phenotype of CC cells by downregulating AURKB expression.

## 4 Discussion

The incidence of CC is increasing annually, with CC being a commonly occurring malignancy in children and adolescents [28]. Along with the basic symptoms of adult CC, chronic abdominal discomfort is a key symptom of CC in children. Therefore, CC in children is often overlooked, resulting in dismal clinical outcomes [29]. Hence, it is imperative to elucidate the mechanism behind CC progression, which would help uncover new approaches to interfere with CC progression, especially in children. Our results demonstrated that ELFN1-AS1 expression was enhanced in CC tissues, and that silencing ELFN1-AS1 inhibited CC progression both *in vitro* and *in vivo*. Furthermore, ELFN1-AS1 silencing led to the upregulation of miR-4270 and resulted in AURKB downregulation, reversing the malignant properties of CC. In conclusion, ELFN1-AS1 regulated the miR-4270/AURKB axis, which resulted in CC progression; thus, targeting ELFN1-AS1 may have diagnostic and therapeutic implications for patients with CC, particularly children.

Previous studies have demonstrated the tumorigenic potential of ELFN1-AS1 in different cancers, including CC. Researchers have shown that siRNAs targeting ELFN1-AS1 disrupted the uncontrolled proliferation and migration of CC cells, thereby interfering with their malignancy [13,16]. A recent study showed that ELFN1-AS1 sponged miR-1250 and upregulated the expression of metastasis-associated protein 1, which promoted the tumorigenic and metastatic behavior of CC cells [30]. Interestingly, ELFN1-AS1 is also regarded as a ferroptosis-related lncRNA that is enriched in cancer- and metabolism-related cascades, such as in CC [31]. Consistent with the earlier reports, we also found that ELFN1-AS1 silencing impeded tumorigenesis *in vitro* and *in vivo,* clearly indicating the pro-oncogenic action of ELFN1-AS1 in CC.

Bioinformatics analysis revealed that miR-4270 is a target of ELFN1-AS1. Their direct binding was further verified in this study using luciferase reporter assays. Their targeted regulatory actions were also observed in RB, where ELFN1-AS1 promoted progression of RB through regulating the miR-4270/SBK1 axis [15]. The authors found that miR-4270 mimics inhibited the proliferation, migration, and invasion of RB cells, showing that it is a tumor suppressor in RB [15]. Surprisingly, miR-4270 plays opposing roles in various cancers [26,27,32]. For instance, miR-4270 was downregulated in gastric cancer and hepatocellular (HCC) tissues as compared to the precancerous tissues, implying a tumor suppressor role [26]. On the contrary, it was significantly upregulated in the blood of breast cancer patients [33]. Furthermore, the rescued expression of p53 via miR-4270 inhibition induced nasopharyngeal carcinoma cells to be more sensitive to irradiation [27]. However, the role of miR-4270 in CC remains unclear. To the best of our knowledge, our findings indicated for the first time that miR-4270 silencing increased CC cell proliferation and motility and triggered apoptotic inactivation. Our results in CC are in line with previous findings in gastric cancer, HCC, and CC, where miR-4270 functions as a tumor suppressor in CC. Mechanistically, miR-4270 silencing abrogated the tumor-suppressive function of ELFN1-AS1 silencing. ELFN1-AS1 knockdown further antagonized the low expression of miR-4270 caused by miR-4270 inhibitor, further supporting the negative regulation of ELFN1-AS1 on miR-4270. Collectively, ELFN1-AS1 sponged miR-4270 and facilitated CC progression.

According to the ceRNA hypothesis, lncRNAs compete for miRNA binding and keep miRNAs away from their target mRNAs, reshaping the mRNA expression landscape of a cell [34]. Using TargetScan analysis, we found that AURKB might be a target of miR-4270. Simultaneously, we used luciferase reporter assays to verify that miR-4270 could target the 3′-UTR of AURKB. We reported the interaction between miR-4270 and AURKB in CC for the first time. RT-qPCR analysis showed that the miR-4270 inhibitor antagonized the loss of AURKB expression in CC cells transfected with si-AURKB. More importantly, the miR-4270 inhibitor restored the antitumor action of si-AURKB in CC cells. Therefore, miR-4270 suppresses CC progression by downregulating AURKB expression. The negative correlation between miR-4270 and AURKB expression further verified the antagonistic relationship between these molecules.

The AURKB gene is located in the locus 17p13.1, comprising seven exons. This gene encodes a member of the Aurora kinase subfamily of serine/threonine kinases. These kinases participate in regulating chromosome alignment and segregation during mitosis and meiosis by associating with microtubules. Convincing data have demonstrated the oncogenic action of AURKB in various cancers, such as gastric cancer [35] and clear cell renal cell carcinoma [36]. AURKB expression has been shown to positively regulate cancer proliferation, metastasis, and drug resistance [35,37–39]. For example, AURKB was found to be associated with the clinicopathological characteristics of gastric cancer, and its high expression activated the Wnt/β-catenin/Myc cascade to induce epithelial–mesenchymal transition [36]. AURKB overexpression results in chromosomal mis-segregation errors, which are hallmarks of cancer, including colorectal cancer [40]. However, little is known about its role in CC. Our functional assays demonstrated that AURKB silencing attenuated uncontrolled cell proliferation and migration. The Bcl-2 family of proteins comprises key regulators of the mitochondria-mediated apoptosis pathway, including the pro-apoptotic protein Bax and the anti-apoptotic protein Bcl-2 [41]. Studies have shown that AURKB inhibited the intrinsic resistance of malignant tumors to autophagy-related death by overcoming Bcl-2 expression [42]. In addition, AURKB inhibited the apoptosis of breast cancer cells by promoting Bcl-2 expression and inhibiting Bax expression [43]. Similarly, this study found that AURKB knockdown reduced the protein level of Bcl-2 and increased Bax expression in CC cells, revealing the pro-apoptotic effect of AURKB knockdown on CC. Our findings provide further evidence of the oncogenic role of AURKB in malignancy. As in RB, AURKB could be a therapeutic target, and targeting it could be a unique therapeutic technique to limit the progression of CC [44]. Therefore, we might provide a novel optimal target for CC therapy.

Our study had several limitations. The regulatory network associated with ELFN1-AS1 in CC may be complex because of the promiscuity of miRNA seed sequences. Therefore, ELFN1-AS1 may interact with different miRNAs and regulate CC progression through another mechanism. Moreover, more clinical samples are required to validate our findings. Furthermore, the downstream effectors or signaling cascades related to AURKB in the CC context requires further investigation.

In conclusion, we established ELFN1-AS1 as an oncogenic lncRNA that promotes CC progression by regulating miR-4270 expression, thereby upregulating AURKB expression. In addition, inhibition of AURKB inhibition could be a potential treatment strategy in CC. As a result, ELFN1-AS1 and AURKB warrant further investigation as an intriguing therapeutic target to reduce the occurrence of detrimental consequences associated with CC tumorigenesis and progression.
